# Standard method for microCT-based additive manufacturing quality control 3: Surface roughness

**DOI:** 10.1016/j.mex.2018.09.004

**Published:** 2018-09-15

**Authors:** Anton du Plessis, Philip Sperling, Andre Beerlink, Oelof Kruger, Lerato Tshabalala, Shaik Hoosain, Stephan G. le Roux

**Affiliations:** a CT Scanner Facility, Stellenbosch University, Stellenbosch, South Africa; b YXLON International GmbH, Hamburg, Germany; c National Metrology Institute of South Africa, Pretoria, South Africa; d National Laser Centre, Council for Scientific and Industrial Research, South Africa

**Keywords:** X-ray tomography, MicroCT, Nondestructive testing, Additive manufacturing, Quality control, Surface roughness

## Abstract

The use of microCT of 10 mm coupon samples produced by AM has the potential to provide useful information of mean density and detailed porosity information of the interior of the samples. In addition, the same scan data can be used to provide surface roughness analysis of the as-built surfaces of the same coupon samples. This can be used to compare process parameters or new materials. While surface roughness is traditionally done using tactile probes or with non-contact interferometric techniques, the complex surfaces in AM are sometimes difficult to access and may be very rough, with undercuts and may be difficult to accurately measure using traditional techniques which are meant for smoother surfaces. This standard workflow demonstrates on a coupon sample how to acquire surface roughness results, and compares the results from roughly the same area of the same sample with tactile probe results. The same principle can be applied to more complex parts, keeping in mind the resolution limit vs sample size of microCT.

## Graphical abstract

**Figure:**
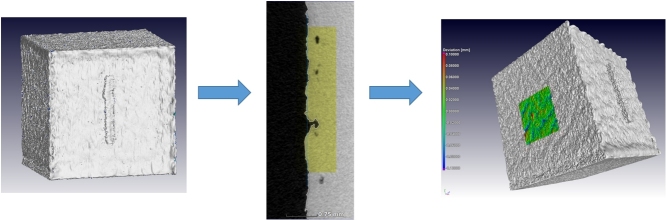

## Introduction

The surface topography of AM parts has been measured by microCT in a number of recent studies of complex parts [[1], [2], [3]]. Despite these efforts no dedicated workflow has been described yet. In this work we propose one standard workflow dedicated to a 10 mm coupon sample. This coupon is already in use for other analysis purposes, as described in previous method papers in our group for detailed porosity analysis [4] and mean sample density [5]. The method has also be demonstrated in a recent review paper on a cylindrical surface [6]. This surface characterization method can be applied to existing scan data, thereby reducing the cost barrier to the adoption of microCT as a method of 3D characterization. This standard workflow aims to support the characterization needs for qualification and standardization in AM.

## Method

The samples were built on a custom built selective laser melting platform within a commercial LENS enclosure. The laser used was an IPG YLS 5000 ytterbium 5 kW fibre laser, wavelength of 1076 nm with a delivery fibre core diameter of 50 μm. The scanner used was an Intelliweld 30 FC V system. Materials used were Ti6Al4V provided by TLS Technik GmbH, gas atomized with particle size 20-60 μm. The base plate material is Ti6Al4V, 150 mm in diameter, approximately 40 mm thick. The hatch parameters used were a power of 3 kW, speed of 3 meters/second, and a 240 μm spot size. The contour scans used a laser power of 1 kW where the hatch-contour distance and the speed was varied to investigate its effect on density (porosity) and surface finish. As this paper specifically focusses on the method of measurement only one sample was evaluated (Table 1).

**Table 1:** Surface area comparison microCT vs tactile probe for 3 × 3 mm side wall selection.

	MicroCT	Tactile probe
Sa	29.6 μm	36.9 μm

As described previously, a standard coupon test sample of 10 × 10 × 10 mm is suggested for this test. This size allows a reasonably high scan resolution while allowing a large enough size for practical purposes. Tactile probe measurements were performed using a Taylor Hobson system with 2 μm probe radius, at the National Metrology Institute of South Africa. The probe acquired points along the movement direction at 3 μm with 1000 points, and each line of points spaced 1 μm from the next for 3000 lines, covering a total of 3 × 3 mm.

Typical laboratory microCT is used as for example found in [7], with parameters optimized according to the guidelines in [8]. MicroCT scan settings of 200 kV, 70 uA, with 0.5 mm beam filter are used, with image acquisition of 500 ms per image, 2400 step positions in a full 360 degree rotation. The voxel size is 15 μm. At each step position, the first image is discarded and two subsequent images averaged. The total scan time is just under 1 hour. When sample setup, machine warmup, background correction and reconstruction is included this should be possible in almost any system in 2 hours total. The reconstruction is done using a strong beam hardening correction factor without any image de-noising.

The data is the analysed in Volume Graphics VGStudioMax 3.1. The image processing is described here as also described previously [4,5] (and hence does not have to be repeated if done for other analyses). The first part described the removal of the exterior air from the data set, but including all material and air (pores). This is done by first applying a basic surface determination, following by creating a region of interest (ROI) from this surface. A region growing tool is then used with high tolerance on the air outside the part, while the option is selected for “avoid other visible ROIs”. This selects all exterior air up to the edge of the part. Inverting this selection allows to select the entire part including its voids. A new advanced surface determination function is then applied, using this ROI selection as a starting contour. In this way the local optimization is performed on the exterior surface, allowing the best subvoxel precision on the surface location.

**Fig. 1:**
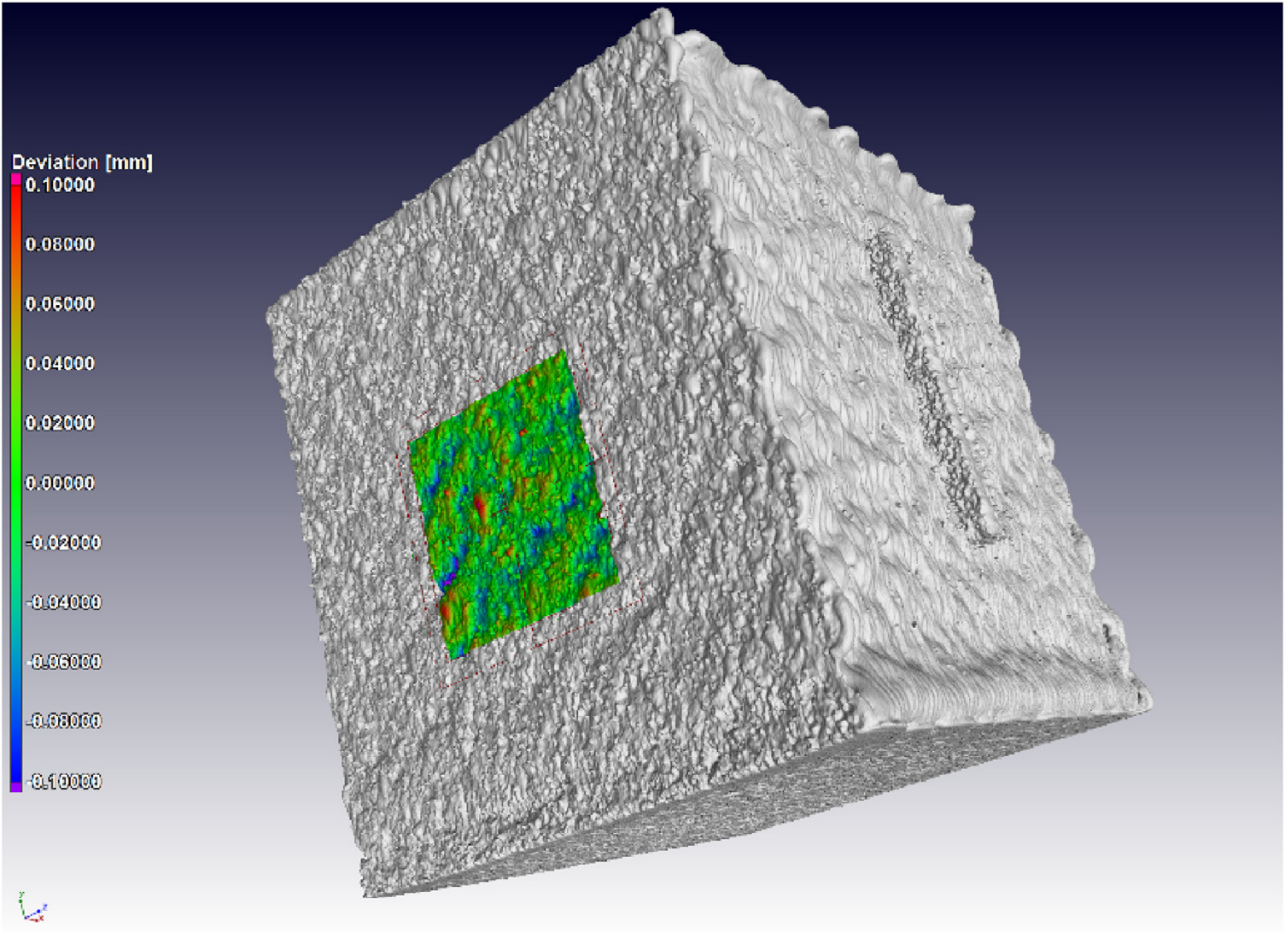
MicroCT surface topography, for selected 3 × 3 mm area on vertical side wall of cube.

**Fig. 2:**
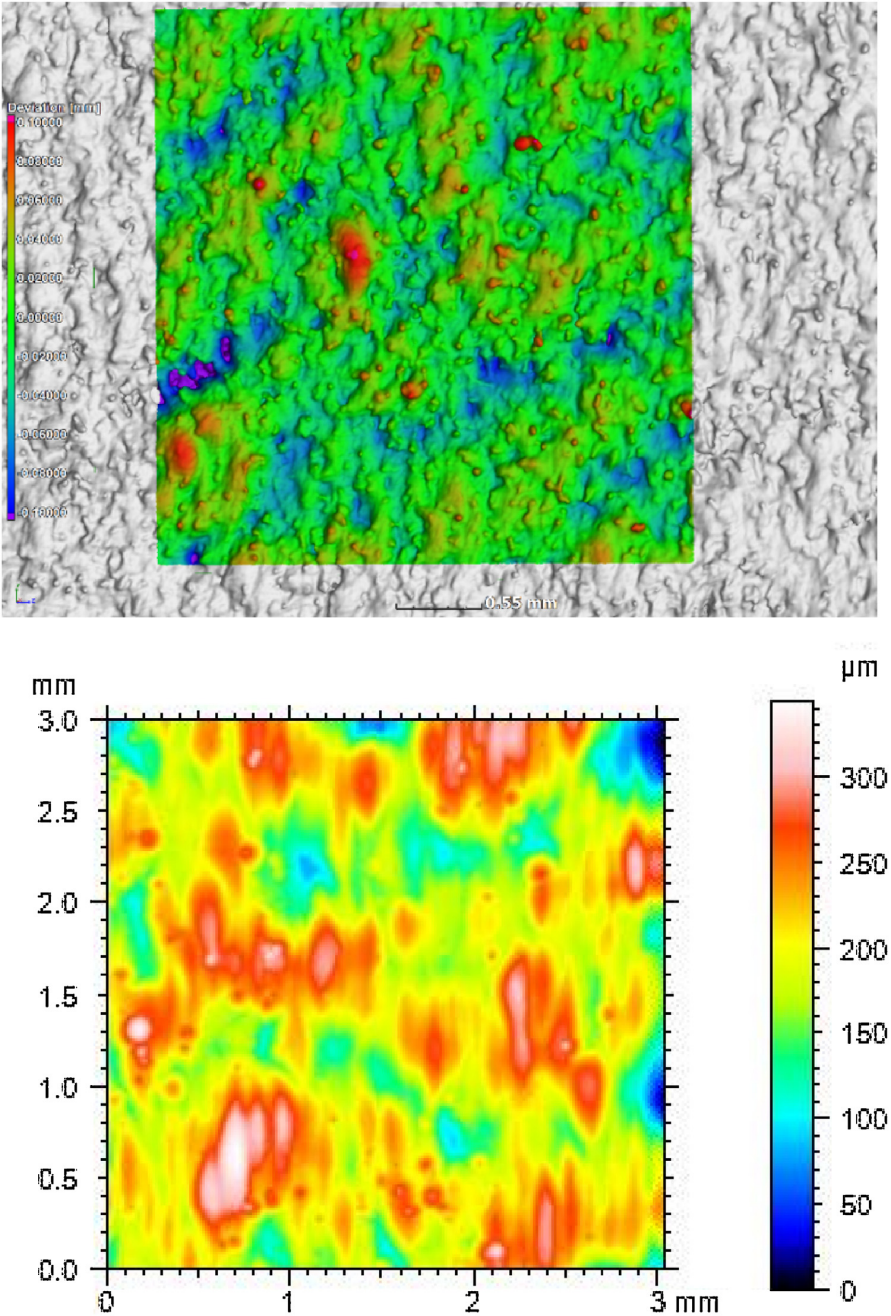
3D image of topography from (a) microCT vs (b) tactile probe (of a 3 mm square area on the vertical side wall). The differences in colour coding may be due to slight differences in the area of interest, and differences in sampling.

For surface roughness measurement, the area to be analysed must be selected as a region of interest (ROI) eg. (3 mm x 3 mm x 3 mm) and preferably coloured to visualize its location on the surface. Then a best-fit plane (geometry element) must be selected surrounding this area of interest, by selecting approximately 15 points along each edge of the square-selected region for analysis. This is described for a flat surface but any geometrical feature can be analysed, such as a rod geometry, in the same way. Once this is selected, a mesh is created from the geometry element: right click on the plane select “Create=>Mesh from geometry element(s)”. A nominal-actual deviation is then calculated between the best-fit plane (mesh from geometry element) and the actual surface in the region of interest (the analysis takes places in the ROI). Right click on the ROI of the region to be analysed, select “Create => Nominal/actual comparison”. This colour-map shows the topography and statistical information shows the deviation histogram and cumulative histogram data. The result is shown in Fig. 1, with the colour map on the vertical side wall. Fig. 2 shows a closeup and direct comparison of the same area mapped by microCT and tactile probe.

The microCT data can be extracted to a CSV file in the format of two columns representing the deviation values. The deviations are from minimum (negative values) to maximum (positive values). The number of surface elements for each deviation value is provided in the form of surface area total per deviation value. Therefore, to calculate Sa values, the absolute values of the deviations must be calculated, multiplied with the surface area corresponding to each such deviation, and the total divided by the total surface area. Since roughness is often quoted in terms of Ra values, for a linear measurement, this can be done in the same way, by downscaling the data or by using a line fit instead of a surface area. Since 3D data allows both types of roughness measurements, the Sa measurement would be preferable in most cases, as it is less prone to variations.

**Fig. 3:**
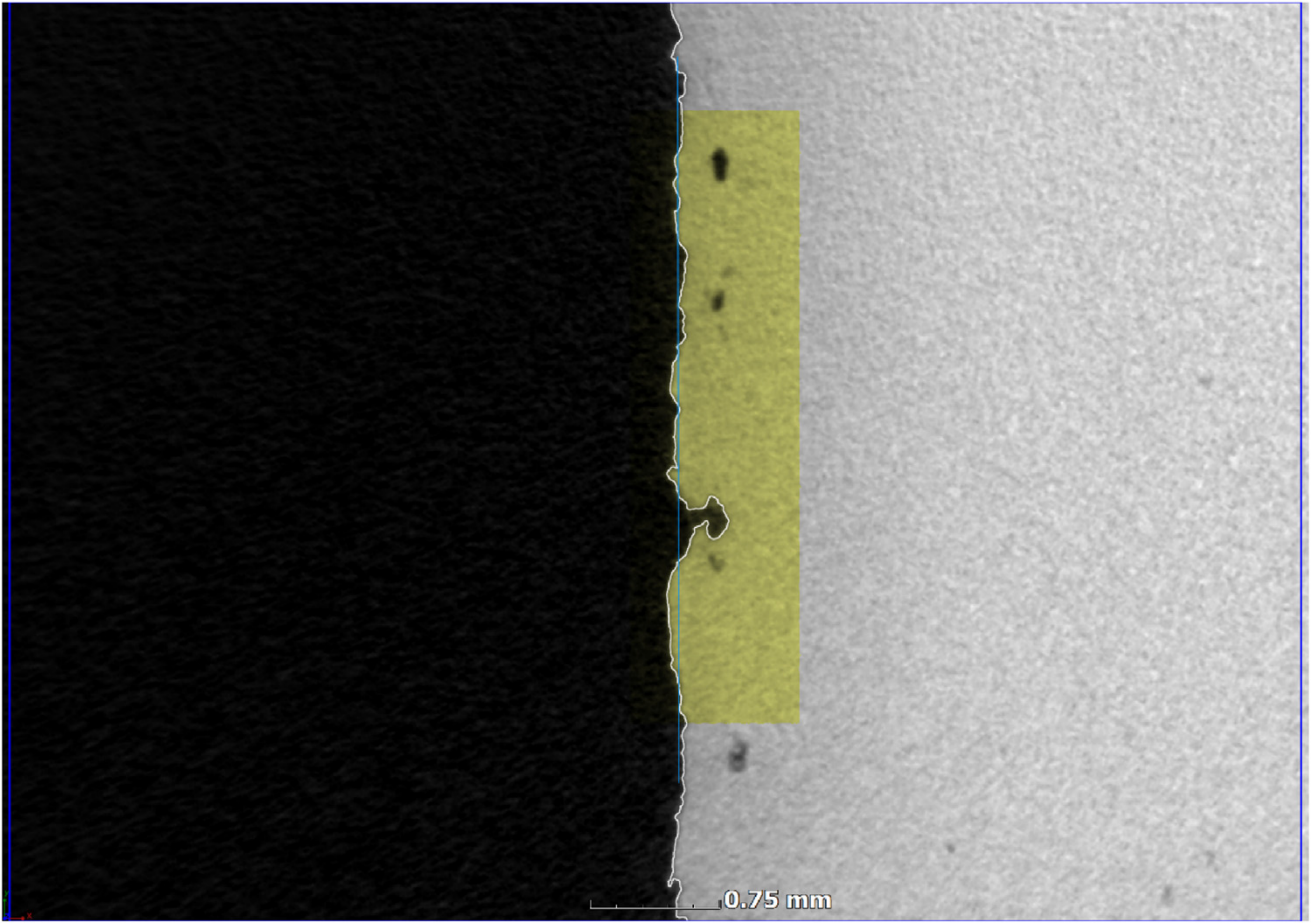
MicroCT topography measurement includes open connected subsurface porosity.

The microCT value is slightly lower than the tactile probe values, which may be due to a slightly different area selection, and differences in sampling steps used. Nevertheless the images demonstrate a good overall comparison for bulk roughness and the method demonstrated here can easily be reproduced. One advantage of using microCT is the additional image information that can be obtained, see for example the deep open surface porosity detected by microCT which is missed by tactile probe in Fig. 3. The light blue line indicates the best-fit plane while the white line indicates the microCT-determined surface of the part. The yellow area is the selected region for analysis (3 × 3 mm on surface, with some depth to include all possible roughness).

## Conclusion

A method was described for surface roughness / topography analysis by microCT on a cube-shaped coupon sample of 10 mm. The method holds promise for improving as-built surface roughness by optimizing process parameters, and the method will find more use when used in combination with other analyses such as porosity analysis, from the same scan data. The coupon sample suggested allows 15 μm voxel size with good quality scan data – the method can be modified to suit different applications such as more complex parts with non-flat surfaces and internal surfaces.

## Appendix A Supplementary data

A video is included which demonstrates the image analysis workflow step by step. The analysed volume data for one sample is available and will be published online and made freely accessible.
