# Supplementary material for: Standard method for microCT-based additive manufacturing quality control 3: Surface roughness
Source: MethodsX. 2018 Sep 15;5:1111–6. doi: 10.1016/j.mex.2018.09.004 (PMC6168927; doi:10.1016/j.mex.2018.09.004)
Supplement: Supplementary file 1 [file mmc1.docx]

**Supplementary material**

A video is included which demonstrates the image analysis workflow step by step. The analysed volume data for one sample is available and will be published online and made freely accessible.
